# Advanced hemostasis in axillary lymph node dissection for locally advanced breast cancer: new technology devices compared in the prevention of seroma formation

**DOI:** 10.1186/s12893-018-0454-8

**Published:** 2019-04-24

**Authors:** Claudio Gambardella, Guglielmo Clarizia, Renato Patrone, Chiara Offi, Claudio Mauriello, Roberto Romano, Marco Filardo, Alessandra Conzo, Alessandro Sanguinetti, Andrea Polistena, Nicola Avenia, Giovanni Conzo

**Affiliations:** 1 Division of General and Oncologic Surgery - Department of Traslational Medical Sciences, University of Campania “Luigi Vanvitelli”, Via Sergio Pansini 5, 80131 Naples, Italy; 2 Endocrine Surgery Unit, University of Perugia, Piazza dell’Università, 06123 Perugia, Italy

**Keywords:** Advanced breast cancer, Axillary lymphnodes dissection, Advanced hemostasis device, Seroma

## Abstract

**Background:**

Breast cancer is the most frequent neoplasm in women. Axillary lymph nodes dissection represents the treatment of choice in locally advanced breast cancer for prognostic and curative purposes. Seroma formation, an abnormal collection of fluid in the dead space of the axilla, is described in Literature with a wide range of incidence (3–85%). It is a source of significant morbidity and discomfort. The aim of the study is to compare the different haemostasis devices used in breast surgery, investigating the eventual superiority of an instrument among the others in terms of intraoperative and postoperative outcome, especially of seroma formation.

**Methods:**

Clinical cases of female patients undergone axillary lymph nodes dissection for local advanced breast cancer between January 2013 and July 2017 at the Surgery Unit of University of Campania “Luigi Vanvitelli” were retrospectively reviewed. Patients were divided into four groups, according to device utilized during surgery: Electrocautery, Harmonic Scalpel, LigaSure and Thunderbeat. All patients underwent II level axillary lymph nodes dissection associated to radical mastectomy or quadrantectomy.

**Results:**

One hundred consecutives patients were enrolled in the study. Intra-operative blood loss resulted statistically significant different (*P* < 0,01) between the Electrocautery group (94,7 ml) and the Thunderbeat group (57,2 ml), while the Harmonic Scalpel group and the Ligasure group, despite presented a lower amount of blood loss, did not differ significantly. Drainage volume resulted significantly lower (*P* = 0,002) in the comparison between the Electrocautery group and the Thunderbeat group; the Ligasure group and Harmonic Scapel group showed no difference between them and Electrocautery group. About the seroma formation, the Electrocautery group resulted affected by the highest seroma formation rate (64%). Seroma incidence in Harmonic Scalpel group was 24%, in Ligasure group was 44%, while Thunderbeat group showed the lowest presentation of seroma with 16%.

**Conclusions:**

In patients affected by breast cancer requiring axillary lymphnodes dissection, the use of advanced hemostasis devices is highly desirable. Among the non-traditional tools, Thunderbeat resulted to be superior in terms of reduction of intra-operative blood loss and post-operative drainage output, moreover associated to a substantial reduction of postoperative seroma incidence.

## Background

Breast cancer is the most frequent neoplasm in women, with an incidence of fifty thousand new cases diagnosed in Italy in 2017 [1].

Oncological treatment for breast cancer experienced substantial modifications in the past decades, tending to a less invasive approach. The introduction of new screening programs permits to diagnose breast cancer in earlier stages [2, 3], allowing a conservative surgery for tumor stages I-II, associated to sentinel lymph-node biopsy (SLNB), in case of clinically and imaging negativity of Axillary Lymph Nodes (ALN) [2, 4]. Nevertheless, axillary lymph node dissection (ALND) is still considered the gold standard approach in association with radical mastectomy (RM) either quadrantectomy (Qu), for the treatment of locally advanced breast cancer with positive lymph nodes, determined by fine needle cytology (FNC) or a core-needle biopsy, or in selected cases of stage IV tumors [1].

Recent guidelines from the US National Comprehensive Cancer Network describe locally advanced breast cancer as the American Joint Committee on Cancer stage III breast cancer. The definition includes breast cancer that fulfils any of the following criteria in the absence of distant metastasis:

- Tumours more than 5 cm in size with regional lymphadenopathy (N1–3)
- Tumours of any size with direct extension to the chest wall or skin, or both (including ulcer or satellite nodules), regardless of regional lymphadenopathy
- Presence of regional lymphadenopathy (clinically fixed or matted axillary lymph nodes, or any of infraclavicular, supraclavicular, or internal mammary lymphadenopathy) regardless of tumour stage [4].

ALND is associated to different postoperative complications, such as lymphorrhea, seroma formation, lymphedema and functional limitation of the shoulder and of the upper limb. In particular, post-operative seroma formation, a collection of fluid in the dead space of the axilla, is described in Literature with an extremely wide range of incidence, from 3 to 85% [5, 6].

Despite many surgeons consider seroma as a mere post-operative side effect, this complication leads to prolonged hospital stay, discomfort and delayed wound healing. Therefore, seroma formation may implicate delayed beginning of adjuvant therapy, affecting oncological outcomes [7, 8].

Risk factors and physiopathology of post-operative seroma are still matter of debate, but the main hypotheses are the cellular damage by thermal effect, and the incomplete vessels and lymph ducts obliteration during the dissection [9, 10].

Many procedures have been proposed in order to reduce the seroma formation (physiotherapy, external compression and use of pharmacological aids such as hemostatic biological adhesives), but none of them produced effective and definitive results [11, 12].

Drain placement is nowadays the only valid method to reduce and treat seroma formation, but on the other hand it leads to discomfort, pain, functional limitation of the arm and its prolonged maintenance may be a cause of infection [12].

The utilization of new devices, widely used in laparoscopic surgery, providing a lower cellular damage and a better vessel sealing and hemostasis, could reduce seroma formation and improve post-operative healing, allowing an earlier beginning of adjuvant therapy. Electrocautery (EC) is an efficient device for ALND surgery, due to an easy manageability and a successful hemostasis, but the high emanated heat strongly affects the incidence of seroma [13].

Introduced in 1990, the Harmonic Scalpel™ (HS) (Ethicon, Somerville, NJ) is a system that allows cutting and hemostasis without the application of electrical energy to the patient. It is based on mechanical energy at a high frequency of 55.5 kHz and it have long been proven to decrease complications and operative time in both open and laparoscopic surgery. Mechanical energy, transmitted by an active blade, results in collagen molecules within the tissue denaturation, generating coagulum with lower thermal injury (< 150 °C) compared to EC. The direct application of ultrasound produces surgical dissection and hemostatic effect, with obliteration of vessels up to 6 mm in diameter [14–16].

LigaSure™ (LS) (Covidien, CO, USA) is an electro-thermal bipolar vessel-sealing system, providing hemostasis by a combination of pressure and electro-thermal energy. This device allows cutting as well as ligation of blood vessels up to 7 mm in diameter, and its application is described in Literature for thyroidal, urological, gynecological and colorectal procedures [17].

Thunderbeat™ (TS) system (Olympus Medical Systems Corp., Tokyo, Japan) is a multifunctional device, which integrates both ultrasonic and advanced bipolar energy in a single instrument, taking advantage of both kinds of energy, and realizing rapid tissue cutting and reliable vessel sealing [18].

The aim of the current study is to compare the different haemostasis devices used in breast surgery, investigating the eventual superiority of an instrument among the others in terms of intraoperative and postoperative outcome, especially of seroma formation.

## Methods

### Study design

Clinical cases of female patients undergone ALND for breast cancer between January 2013 and July 2017 at the General and Oncological Surgery Unit of University of Campania “Luigi Vanvitelli” (Naples - Italy), were retrospectively reviewed. The diagnosis was reached via fine needle cytology (FNC) or core biopsy. All patients had pathologically confirmed ALN metastases. Preoperative written consent was obtained from all participants. Patients with distant metastases, patients underwent neoadjuvant chemo-radiotherapy, patients presenting, at the time of surgery, blood clotting or immune system alterations, patients receiving anticoagulant treatment, and patients undergone previous surgeries on axilla or breast surgeries, were not included in the study. Patients were divided into four groups, according to device utilized during surgery: Electrocautery (EC), Harmonic Scalpel (HS), LigaSure (LS) and Thunderbeat (TB). The Authors included the first 25 subsequent procedures of each group in chronological order, in order to obtain comparable data. Pre-operative, intra-operative and post-operative data of every group were collected from patients’ medical records.

### Management

Before surgery, all patients received antibiotic prophylaxis. All patients underwent II level ALND, with extension to III level in case of macroscopic metastatic presence at II level [1], associated to RM or Qu. All procedures were performed by the same equip experienced in breast surgery according to standardized surgical technique described in Table 1. At the end of every procedure, a closed suction Redon drain was placed in the axilla cavity. In case of mastectomy, a single close-suction drain with a long tip was used to drain both breast cavity and axilla. After surgery, drain output was recorded daily and the drain was removed when the output was less than 30 ml/day. Possible complications were recorded daily in the medical records as well. Seroma collection was clinically recognized, measured and drained by ultrasound (US) guidance, and the amount of serum was recorded. At the discharge, patients’ follow up consisted of clinical evaluation at the day 7–15-30, and eventual complications were also recorded.

**Table 1:** Surgical technique

Surgical Technique	Description
Radical Mastectomy (RM)	Elipsoid incision including the skin portion to be removed. Preparation of the posterior and the anterior strip of skin, followed by breast gland removal in toto, including the neoplasm, with the respect of pectoral fascia.
Quadrantectomy (Qu)	Diamond shape incision, including the tumor cutaneus projection in the middle. Removal of the breast gland until the fascial plan of the underlying muscle. The lateral thickness of removed healthy gland must be at least 1 cm from the neoplasm
Axillary Lymph Node Dissection (ALND)	Incision up the skin projection of the large dorsal muscle (extension of the ellipsoid excision when associated to RM, separate excision when associated to Qu). Preparation of the posterior and anterior strip of the skin, lax tissue removal until the axillary vein, section of intercostobrachial nerve. Identification of the dorsal bundle (arthery, vein, nerve) and the thoracic nerve (N. of Bell) that must be preserved (I level). Expostion and section of the pectoralis minor, in order to access the underlying lymphonodes (II level). Identification of the subclavian tendon and subclavian vein, exciding the lax tissue until the medial edge of the pectoralis minor previously sectioned (III level)

### Statistical analysis

Continuous variables were described as median (age, drainage volume) or mean (Intraoperative times, intraoperative blood loss, drainage removal) and range, while categorical variables were described as number of cases and percentage. Intraoperative blood loss and drain volume were analyzed with normality test of Kolmogorov-Smirnov. Paired t-test was performed to compare the variable intraoperative blood loss, while we performed Test of Proportions for the variable “seroma”. Moreover, the Authors performed the non-parametric 2 independent samples, Kolmogorov-Smirnov test to analyze the statistical difference of the drain volume. Statistical significance was considered in case of *P* value < 0,05. Statistical analysis was performed with SPSS version 23 (SPSS©, Chicago, IL, USA).

## Results

**Fig. 1:**
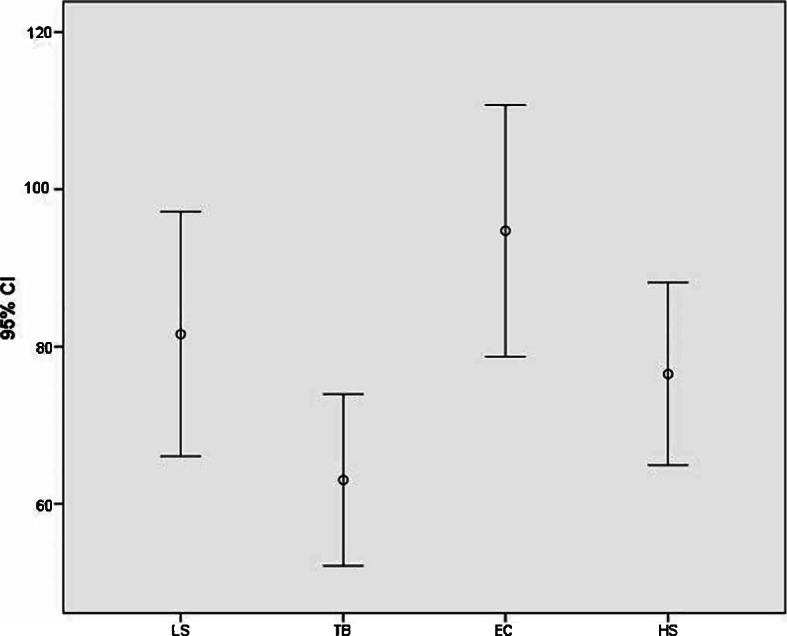
Error bar for graphical representations of the variability of data. Each Error Bar of Intraoperative Blood Loss is constructed using 95% CI of the means

**Fig. 2:**
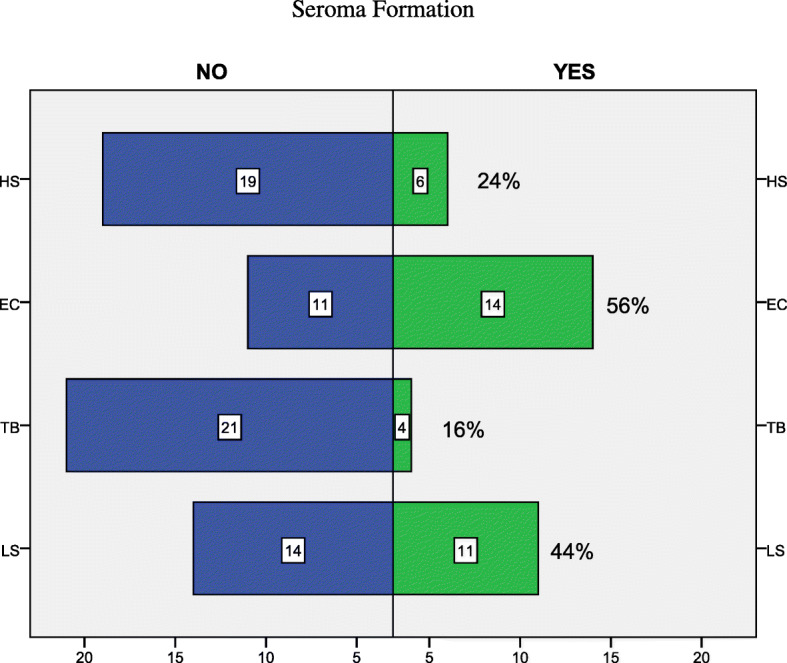
Relative frequency percentage referred to upcoming seroma of individual instrument. LS (Ligasure); TB (Thunderbeat); EC (Electrocautery); HS (Harmonic Scalpel)

From January 2013 to July 2017, 100 consecutives female patients were enrolled in the current study. Patients were retrospectively assigned to one of the four groups according to the device used during surgery. Demographic data about every group are reported in Table 2. Regarding surgery, intra-operative time was lower in the EC group (137,5 min for a TM + ALND, 88 min for a Qu + ALND) than in all the other groups. (Table 3) Comparing data about intra-operative blood loss for the ALND surgical time, it resulted a statistically significant difference (*P* < 0,01) between the EC group (94,7 ml) and the TB group (57,2 ml). (Fig. 1) The HS group and the LS group, despite presented a lower amount of blood loss, did not differ significantly compared to EC. (Table 3) The number of harvested lymph nodes instead was similar for all four groups. Concerning post-operative output, drainage volume resulted significantly lower (*P* = 0,002) in the comparison between the EC group and the TB group. (Table 4) Regarding post-operative complications, the EC group resulted affected by the highest seroma formation rate (64%), while its incidence in HS group was 24% of the cases, in LS group was 44% of the cases, and TB group showed the lowest presentation of seroma with 16% of the treated patients. (Table 4, Fig. 2) Specifically, the TB group resulted significantly different with EC group (*p* = 0,004) and LS group (*p* = 0,035). In the present series, TB application resulted also in a lowest rate of lymphedema presentation. (Table 4).

**Table 2:** Demographic data, tumor location, patients’ comorbidities – EC (Elettrocautery); HS (Harmonic Scalpel); SM (LigaSure); TB (ThunderBeat); UEQ (Upper-External Quarter); UIQ (Upper-Internal Quarter); LEQ (Lower-External Quarter); LIQ (Lower-Internal Quarter)

	EC	HS	LS	TB
Patients, *n*.	25	25	25	25
Age – median [range], yrs	52 [33–70]	49 [35–73]	45 [27–61]	54 [37–68]
Laterality – right breast, *n*. (%)	15 (61%)	18 (72%)	11 (44%)	14 (56%)
Tumor location – UEQ, *n*. (%)	13 (52%)	14 (56%)	15 (60%)	11 (44%)
Tumor location – UIQ, *n*. (%)	6 (24%)	5 (20%)	6 (24%)	7 (28%)
Tumor location – LEQ, *n*. (%)	4 (16%)	4 (16%)	4 (16%)	3 (12%)
Tumor location – LIQ, *n*. (%)	2 (8%)	2 (8%)	0 (0%)	4 (16%)
Body mass index - median [range], Kg/m2	27,1 [19–35]	26 [20,5–34]	24,5 [18,6-30,2]	26,8 [22–31,5]
Comorbidities – hypertension, n. (%)	5 (20%)	6 (24%)	4 (16%)	5 (20%)
Comorbidities – Diabetes, *n*. (%)	1 (4%)	1 (4%)	1 (4%)	0 (0%)

**Table 3:** Type of surgery and intra-operative data - EC (Elettrocautery); HS (Harmonic Scalpel); SM (LigaSure); TB (Thunder Beat); RM (Radical Mastectomy); Qu (Quadrantectomy)

	EC	HS	LS	TB
Radical Mastectomy + ALND – *n*. (%)	8 (32%)	11 (44%)	9 (36%)	9 (36%)
Quadrantectomy + ALND – *n*. (%)	17 (68%)	14 (56%)	16 (64%)	16 (64%)
Intraoperative time (RM + ALND) – mean [range], min.	137,5 [100–170]	159,5 [140–190]	150,3 [118–220]	147,8 [10–205]
Intraoperative time (Qu + ALND) – mean [range], min.	88 [60–100]	90,3 [55–110]	91 [50–108]	99,4 [60–135]
Intra-operative blood loss – mean [range], mL	94,7 [32–150]	76,5 [30–129]	81,6 [23–135]	57,2 [22–103]
Lymph nodes harvested - mean [range], *n*.	14,9 [7–26]	15,4 [6–28]	15 [8–28]	14,3 [6–26]

**Table 4:** Post-operative data and complications - EC (Elettrocautery); HS (Harmonic Scalpel); SM (LigaSure); TB (Thunder Beat)

	EC	HS	LS	TB
Drainage volume - median [range], mL	640 [30–720]	600 [30–650]	600 [90–750]	520 [60–670]
Drainage removal – mean [range], days	5,6 [2–10]	5,1 [2–9]	6[3–9]	5,52 [3–11]
Hospital stay – median [range], days	6 [3–11]	5,6 [3–10]	6,3 [3–11]	5,9 [4–13]
Seroma - *n*. (%)	14 (64%)	6 (24%)	11 (44%)	4 (16%)
Lymphedema – *n*. (%)	2 (8%)	2 (8%)	1 (4%)	0 (0%)
Wound infection – *n*. (%)	1 (4%)	0 (0%)	1 (4%)	1 (4%)

## Discussion

Radical breast surgery and ALND indications experienced a substantial reduction for the widespread of conservative surgery associated to SLNB [4, 19–21], which contributed to an effective and less invasive treatment of early-stage breast cancers [4, 22, 23]. Nevertheless, radical or conservative breast surgery, associated to ALND and followed by adjuvant therapy remains the main approach for treating advanced-stage breast cancer with pathologically confirmed lymph node involvement.

Seroma formation is considered by many Authors as a simple post-operative side-effect [9], but on the other hand, several researches underlined how seroma formation leads to a delay in wound healing, implying a delay in adjuvant therapy beginning [24, 25]. Thus, it is clear the paramount importance of preventing post-operative seroma formation, that could be considered the most frequent complication after breast cancer surgery.

Unfortunately, nowadays definitive etiology and physiopathology of seroma formation are still unknown, despite different hypothesis were expressed, such as acute inflammatory reaction following surgical trauma, response to increased fibrinolytic activity in serum and lymph, reduction of fibrinogen levels in plasma [9, 10, 26–28]. Therefore is extremely hard to achieve an evidence-based therapy.

Patient’s high body mass index, hypertension, preoperative radiation, tumor size, extended breast surgery, extended axillary lymph node involvement and the use of electrocautery have all been related to seroma formation [29–36].

Various attempts have been proposed in order to reduce seroma formation, such as external compression dressing [37], drainage placement [38–41], usage of fibrin glue [42, 43], flap fixation and obliteration of the dead space [44], unfortunately with inconsistent results.

Several Authors proposed that post-operative seroma is due to an unsuccessful closure of lymphatic and arterial vessels of the breast and the axilla, in association to thermal damage [45, 46]. Considering these hypotheses, the application of EC in association with vessels sealing by laces (standard technique) [47, 48], would be the principle cause of seroma formation, whereas the smallest vessels would not be bound, and the EC’s overheating would generate thermal cellular damage [49]. For this reason, different Authors started proposing the use of alternative devices, in order to obtain a better binding and a reduced thermal damage. It is worldwide accepted that the use of advanced hemostasis devices reduces the use of clips and clamps and ties maneuvers, with lower thermal trauma compared to EC. They are easy, manageable instruments, needing a short learning curve [16].

HS, based on ultrasound energy technology, was proposed as an alternative device in the ALND. About its application, opposing results are present in Literature: a meta-analysis from 2012 by Currie et al. [50] implied that no difference was present between HS and EC as regard to intra-operative time, blood loss, drainage volume and seroma formation. Conversely, subsequent studies, reported significant reduction in seroma formation [51–53], reduced intra-operative time [54], reduced intra-operative blood loss [55] and reduction in drainage output [56]. On the other hand, Militello et al. [14], Selvendran et al. [57] and Manjunath et al. [58] showed how the application of HS did not reduce the incidence of seroma after breast surgery. Furthermore, despite the alternative results, various studies underline the high costs for the usage of HS [52, 57, 58].

Other Authors proposed LS to be the superior device in breast surgery and ALND, but also in these cases, the results are not conclusive. In fact, even if pro studies underlined how the LS utilization led to reduced intraoperative time [59–61], reduction of drainage output [60, 62], conversely, several Authors showed no substantial difference when compared to EC [63–66], underlining also a not significant reduction of the cost of hospitalization [67].

No studies in Literature proposed the utilization of TB for breast surgery, which is nowadays frequently described in thyroid and laparoscopic surgeries, showing proficient results [16, 67]. TB is the last synthesis device introduced in surgery which exploit the combination of high-frequency bipolar energy and ultrasound energy. This synergy allows the synthesis and cutting of vessels up to 7 mm in diameter with minimum radial heat dispersion and a short time of application. Specifically, the reduction of the thermal damage to the surrounding tissues, and subsequently of the inflammatory processes, among the main pathogenetic hypothesis of the common side effects of breast surgery, could led to a sharp improvement of postoperative outcomes.

To the best of our knowledge, this is the first comparative study on breast and axilla surgery involving and comparing all the three major advanced hemostasis devices vs EC. In our series HS and LS did not show any significant difference in terms of intra-operative blood loss (Fig. 2) and post-operative variables (drainage output, seroma formation) compared to other devices, confirming the conflicting outcomes present in Literature.

Otherwise, in the comparison between TB and EC, the Authors founded a statistically significant reduction for intra-operative blood loss and post-operative drainage output (*p* < 0,05), associated to a substantial reduction of seroma incidence (16% for TB vs 64% for EC *p* = 0,004). These results could be due to a more effective closure of the vessels, given by the combination of ultrasonic and advanced bipolar energy, confirming the theory of lymphatic origin of seroma [9, 27]. Moreover, the results presented by the Authors on safety and efficacy underlined that the new generation devices did not affect the number of lymphnodes harvested and the incidence of postoperative bleeding or neural injury.

Concerning TB’s costs for breast surgery, a cost-benefit analysis is still matter of intense debate. Nevertheless, a review of 2014 by Shabbir et al. showed a lower cost for TB compared to HS and LS [16].

Our study presents some limitations, such as the limited number of patients involved and the retrospective nature, but the Authors tried to reduce these biases by selecting consecutives clinical cases following a chronological order. Moreover, should be considered that in case of radical mastectomy, breast and axillary cavities are interconnected, and therefore, the drain output is certainly higher compared to quadrantectomy alone. Nevertheless, the results observed could be a starting point to proceed with a prospective study on the use of advanced hemostasis devices for breast surgery.

## Conclusion

In patients affected by breast cancer requiring ALND, the use of advanced hemostasis devices is highly desirable. Among the non-traditional tools, TB resulted to be superior in terms of reduction of intra-operative blood loss and post-operative drainage output, moreover associated to a substantial reduction of postoperative seroma incidence. LS and HS presented intraoperative and postoperative outcome essentially superimposable among them, even if LS was affected by a sharply high incidence of seroma formation. Further prospective randomized controlled studies are needed in order to evaluate the clear advantages of TB in breast cancer, considering also a detailed analysis of costs.

## Abbreviations

ALN: Axillary Lymph Nodes
ALND: Axillary Lymph Nodes Dissection
EC: Elettrocautery
HS: Harmonic Scalpel
LS: Ligasure
Qu: Quadrantectomy
RM: Radical Mastectomy
SLNB: Sentinel Lymph Node Biopsy
TB: Thunderbeat

## Author information

Gambardella Claudio and Mauriello Claudio are PhD student in Medical, Clinical and Sperimental Sciences at Univerity of Campania “Luigi Vanvitelli” – Naples, Italy.
